# Robust Multi-Modal Image Registration for Image Fusion Enhancement in Infrastructure Inspection

**DOI:** 10.3390/s24123994

**Published:** 2024-06-20

**Authors:** Sara Shahsavarani, Fernando Lopez, Clemente Ibarra-Castanedo, Xavier P. V. Maldague

**Affiliations:** 1 Computer Vision and Systems Laboratory (CVSL), Department of Electrical and Computer Engineering, Faculty of Science and Engineering, Université Laval, Quebec City, QC G1V 0A6, Canada; clemente.ibarra-castanedo@gel.ulaval.ca; 2 TORNGATS Services Techniques, 200 Boul. du Parc-Technologique, Quebec City, QC G1P 4S3, Canada; fernando.lopez-rodriguez.1@ulaval.ca

**Keywords:** IR and VIS image registration, IR and VIS image fusion, feature-points threshold, non-maximum suppression, homography estimation, template matching, self-supervised learning

## Abstract

Efficient multi-modal image fusion plays an important role in the non-destructive evaluation (NDE) of infrastructures, where an essential challenge is the precise visualizing of defects. While automatic defect detection represents a significant advancement, the determination of the precise location of both surface and subsurface defects simultaneously is crucial. Hence, visible and infrared data fusion strategies are essential for acquiring comprehensive and complementary information to detect defects across vast structures. This paper proposes an infrared and visible image registration method based on Euclidean evaluation together with a trade-off between key-point threshold and non-maximum suppression. Moreover, we employ a multi-modal fusion strategy to investigate the robustness of our image registration results.

## 1. Introduction

The fusion of infrared and visible imaging techniques in inspecting industrial infrastructure has emerged as a robust strategy, facilitating an efficient and comprehensive assessment [1]. The distinctive insights provided by both infrared and visible images contribute to the identification of diverse defects and anomalies within structures [2,3,4]. Nevertheless, understanding a fully automated defect detection system capable of accurately pinpointing, assessing, and comprehensively evaluating the continuity of defects remains a formidable challenge. To address this challenge, image registration and fusion, which is the process of matching two or more images of the same scene acquired from different sensors, with different views and at other times [5], is often proposed.

The primary objective of this paper is to achieve a comprehensive fusion of infrared and visible infrastructure images, thereby extracting detailed information about surface and subsurface structures to enhance the efficacy of infrastructure inspection. The utilization of facilities equipped with both infrared and visible cameras, while promising, presents several challenges that demand careful consideration.

This paper proposes an effective and robust method to obtain a high-precision multi-modal image fusion for enhancing infrastructure images. We concentrate on both improving alignment accuracy and reducing distortions. The main contributions in this work are as follows:

1. One of the initial drawbacks encountered in this project revolves around the disparities in the field-of-view between infrared and visible sensors. Addressing this challenge involves the implementation of a sophisticated multi-scale template matching approach. This technique enables the alignment and correlation of data captured by sensors with differing fields of view, ensuring an accurate information fusion.
2. Furthermore, a critical challenge lies in aligning the images of both modalities to facilitate an optimal fusion of infrared and visible images. To tackle this issue, we propose a novel and robust approach to image registration. This method enhances the feature detection phase and feature matching phase through a trade-off between non-maximum suppression and key-points threshold using a self-supervised auto-encoder for the feature detection and description phase and a graph neural network for the feature matching phase for infrared and visible image registration. By employing this innovative strategy, we aim to achieve precise alignment, ensuring a seamless integration of infrared and visible data, thereby overcoming the challenges associated with sensor misalignment and contributing to the overall success of the image fusion process.
3. Lastly, a robust multi-modal image fusion is employed to fused the image modalities.

This paper is organized as follows. Section 2 presents the related works. Section 3 explains the multi-modal image registration and fusion strategy, which involves multi-scale template matching, an effective and robust image registration for infrared and visible images, and the infrared and visible fusion strategy. Then, Section 4 outlines the conducted comparative experiments on the algorithms of each part, and our final infrared and visible image fusion result is presented. Lastly, we summarize the entire text in Section 5.

## 2. Related Works

Registering multi-modal images, such as infrared thermography and visible spectrum images, remains challenging due to significant geometric distortions and nonlinear intensity variations. The accuracy of image registration plays a crucial role in subsequent applications, such as image fusion [6,7]. Despite these difficulties, integrating such images is crucial for earth observation applications [7]. Fusing these images provides a comprehensive representation of the area. For non-destructive evaluation of infrastructures, using various sensors and fusing images from different modalities offers valuable information about both surface and subsurface conditions, which is essential for maintaining structures.

This section reviews several key image registration methods, categorized into traditional methods and optimization- and learning-based methods. We then introduce our proposed methodology, which is a learning-based method optimized by a trade-off between non-maximum suppression and key-point threshold.

### 2.1. Fundamental Methods

Area-Based Methods: Area-based (or intensity-based) methods use a robust similarity measure to search for the optimal geometric transformation within a predefined template window. This approach, however, requires extensive computation and can become trapped in local optima. Recent advancements in this area include enhancing similarity measures and refining optimization techniques [8].

Improving Similarity Measures: Cole-Rhodes et al. [9] introduced a multi-resolution remote sensing image registration technique using stochastic gradient optimization for the MI similarity metric. This method enhanced the joint MI histogram by using 64 bins, creating a smoother surface and improving the optimization process. The simultaneous perturbation stochastic approximation (SPSA) algorithm was employed, allowing for efficient gradient approximation and expediting the registration process. Chen et al. [10] developed the Generalized Partial Volume Estimation (GPVE) algorithm, using a second-order B-spline function to compute MI for multi-temporal remote sensing image registration, effectively addressing interpolation-induced artifacts.

Shadaydeh et al. [11] proposed a weight-based joint histogram estimation method (WJH-MI), where each bin in the joint intensity histogram is computed as the sum of weights associated with pixel intensity values. These weights are defined as the exponential function of the distance image and the normalized gradient image, reducing peaks in the joint histogram caused by background or homogeneous regions and producing a more distinct similarity measurement surface.

Xu et al. [12] addressed the challenge of MI with insufficient image overlap by using the symmetric form of the Kullback–Leibler divergence, known as Jeffrey’s divergence. This measures the difference between image pairs by assessing the “distance” between the joint histogram and the product edge histogram of two images, proving to be less sensitive to scene overlap and more suitable for registering small-size images.

#### Feature-Based Methods

Feature-based methods are robust against rotation, translation, and geometric distortion. However, extracting feature points between two images can be challenging due to intensity differences and the need to remove outliers. Consequently, detecting reliable features and designing appropriate descriptors has become a significant challenge.

In multi-modal medical image registration, structure and shape features are effective similarity measures, outperforming traditional methods. Phase correlation or phase congruency (PC) algorithms given by De Castro et al. [13] and Liu et al. [14] are popular due to their insensitivity to illumination and contrast changes. Xie et al. [15] introduced a method using multi-scale Log-Gabor filters combined with phase congruency to resolve non-linear intensity differences. The authors also proposed an extended phase correlation algorithm based on Log-Gabor (LGEPC) to enhance structural information and minimize the impact of radiation differences [16].

Key Feature-Based Methods: Gong et al. [17] proposed a coarse-to-fine multi-modal remote sensing (MMRS) registration approach combining the Scale-Invariant Feature Transform (SIFT) and Harris methods. The UR-SIFT method introduced by Sedaghat [18] is designed for optical multi-source remote sensing images. Fan et al. [19] presented the Spatial Consensus Matching (SCM) algorithm, which uses an improved SIFT and K-nearest neighbors (KNNs) for initial matching, employing RANSAC for transformation parameter estimation. Huang et al. [20] enhanced feature extraction and matching using the Harris operator for control points, the Canny operator for edge features, and an improved shape context descriptor for matching edge feature distributions within a circular template. Xiang et al. [8] introduced an algorithm for high-resolution optical-to-SAR image registration, incorporating multi-scale ratios of exponentially weighted averages (ROEWA) and multi-scale Sobel operators to generate GLOH-like descriptors for stable registration results.

### 2.2. Optimization- and Learning-Based Methods

#### 2.2.1. Optimization-Based Methods

Improving optimization methods is crucial for enhancing the performance and accuracy of image registration, especially in high-resolution and noise-affected images.

Cross-Cumulative Residual Entropy (CCRE): Hasan et al. [21] and Thevenaz et al. [22] introduced the CCRE measure for multi-modal medical image registration. This measure adapts well to images with varying brightness and contrast, offering increased robustness to noise. The Parson-window optimization method was extended with partial volume interpolation to compute the gradient of the similarity measure, improving both the success rate and accuracy for CCRE-based and MI-based algorithms.

Inverse Combinatorial Optimization: Dame et al. [23] presented an inverse combinatorial optimization method to address the quasi-concave shape of MI, allowing for pre-calculation of derivatives and estimation of the Hessian matrix post-convergence. This approach reduces calculation time and facilitates accurate parameter estimation. Furthermore, a method based on selecting reference pixels significantly reduces computation time, resulting in a fast, accurate, and robust registration process.

Ant Colony Optimization (ACO): Liang et al. [24] and Wu et al. [25] used the ACO algorithm to maximize MI. However, the MI similarity curve often has numerous local optima. Yan et al. [6] proposed a transfer optimization (TO) method to enhance global search capability and reduce the risk of converging to local optima by transferring superior results to another optimizer during the iterative process.

#### 2.2.2. Learning-Based Methods

Recent advancements in deep learning and machine learning have led to the development of learning-based methods for image registration. These methods leverage the power of neural networks to learn complex transformations and similarity measures from data.

Deep Learning for Image Registration: Learning-based methods utilize neural networks to directly predict transformation parameters or similarity measures. These methods can handle complex and nonlinear relationships between images, making them particularly effective for multi-modal and non-rigid registration tasks.

Transfer Learning for Optimization: Yan et al. [6] proposed a transfer optimization (TO) method that combines transfer learning with traditional optimization techniques. By transferring superior results to another optimizer during the iterative process, TO enhances the global search capability and reduces the risk of converging to local optima.

## 3. Materials and Methods

In infrastructure inspection, the fusion of various imaging modalities is imperative for a thorough analysis [26]. This paper focuses on aligning visible and thermal images to facilitate accurate interpretation and visualization to achieve precise multi-modal image fusion.

**Figure 1:**
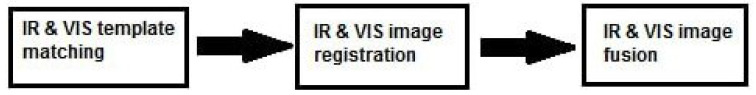
The steps of the proposed method for infrared and visible image fusion.

Figure 1 indicates the steps of the proposed method. The initial challenge addressed in this methodology is the disparate field of view in different image modalities. To this end, the first step involves identifying the shared regions between the two image modalities. It is accomplished by applying multi-scale template matching, enabling the discovery of common areas within the images. Subsequently, a novel multi-modal image registration method is introduced. Accurately aligning images across diverse modalities is crucial, especially in complex infrastructure conditions with poor texture. In the final phase, a visible and infrared fusion strategy is employed to offer complementary information for defect detection, while also illustrate the robustness of the image registration process. The proposed methodology is specifically appropriate to address these issues. This paper, employing visible and infrared thermography images, introduces a comprehensive visualization for infrastructure inspection and employs a fusion strategy to visualize surface and subsurface defects comprehensively. The methodology presented in this paper is implemented to address the intricacies of infrastructure inspection, providing a robust and effective solution for aligning and fusing images from diverse modalities.

**Figure 2:**
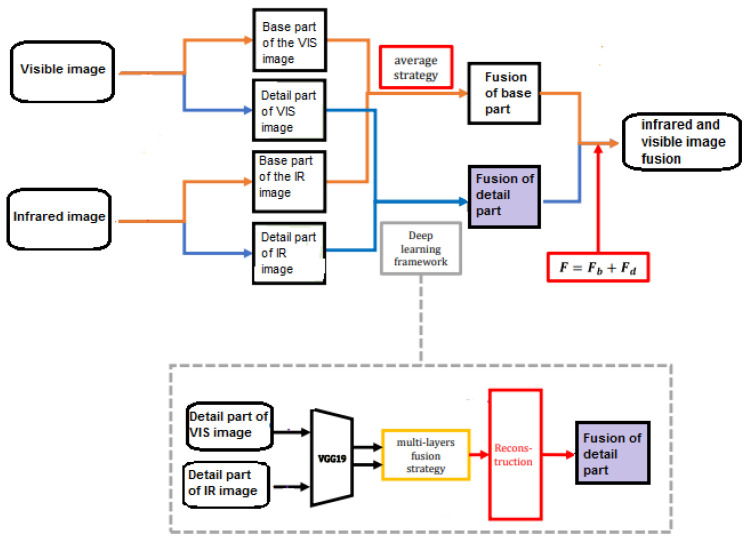
Visible and infrared fusion frame.

Figure 2 illustrates the proposed fusion procedure departing from a visible image and its corresponding thermal counterpart. Notably, thermal and visible images are first cropped if necessary to cover the same area of the scene. In the thermal domain, some details are stored as metadata rather than being fully integrated into the image. The proposed methodology for image registration addresses this discrepancy by precisely identifying the visible image segments corresponding to the thermal representation. This critical prepossessing step aims to determine the overlap between the two image modalities, guiding the subsequent registration process to focus exclusively on the shared components. We optimize the registration for relevance and accuracy by isolating the common elements.

### 3.1. Template Matching for Infrared and Visible Images

Template matching is a critical technique utilized to locate instances of a template image within a larger target image, playing a fundamental role in infrared (IR) and visible (VIS) image registration and fusion [27]. Despite the visual differences between IR and VIS images of the same scene, both share crucial commonality in their edges, which can be effectively utilized for feature identification.

In this section, a comprehensive methodology is presented for template matching appropriate specifically for IR and VIS image fusion applications. This approach employs multi-scale template matching facilitated by edge detection, allowing identification of the infrared image within the visible images and subsequently determining the shared regions between the two images.

Algorithm 1 indicates the steps encompassed by the presented multi-scale template matching algorithm.   

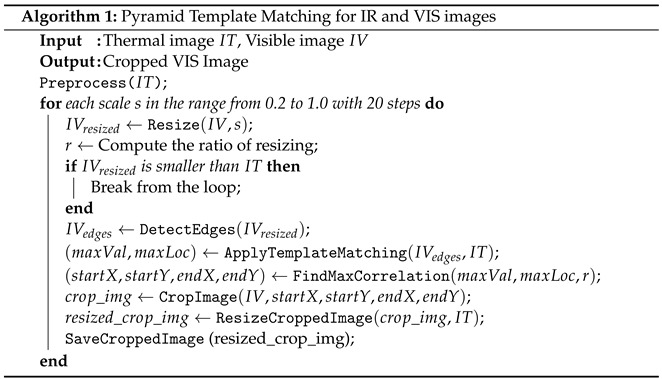


Image Prepossessing: Initially, the input images undergo prepossessing. The thermal image, the template, is converted to grayscale and subjected to edge detection using the Canny algorithm. This crucial step aims to extract edge features from the thermal image, enhancing the discriminative information utilized for subsequent matching.

Multi-Scale Matching: To accommodate scale variations in the visible image, template matching is performed at multiple scales. The visible image is resized to various scales, and template matching is iteratively applied using the Canny-edge-detected thermal image. This multi-scale approach ensures robust matching across different resolutions.

Bounding Box Localization: Upon identifying a match, a bounding box is drawn around the detected region in the visible image. The coordinates of this bounding box are computed based on the scale ratio and the location of maximum correlation. This step facilitates precise localization of the matched region within the visible image.

### 3.2. Infrared and Visible Image Alignment and Registration Block

Obtaining a robust multi-modal image fusion that conveys comprehensive insights from the surface and subsurface of infrastructure represents a paramount endeavor in diverse fields. The success of such fusion critically depends on the meticulous alignment of image modalities, emphasizing the necessity for image registration techniques. The image registration of infrared (IR) and visible (VIS) images becomes especially imperative due to prevalent misalignments arising from variations in sensor and optic focal length. Obtaining rich information about infrastructure is crucial to addressing this misalignment challenge through precise image registration. This section investigates the nuanced landscape of IR and VIS image registration, navigating the complexities of aligning sensors to facilitate multi-modal fusion. Through an in-depth exploration, this research aims to advance image registration methodologies tailored to the intricacies of infrastructure inspection, thereby enhancing the accuracy and reliability of subsequent analyses in diverse application domains.

#### 3.2.1. Feature Detection and Matching Block for Multi-Modal Images of a Single Scene

This section presents a feature detection and description technique. Employing an auto-encoder to enhance the efficiency of feature point detection and description [28,29]. We used a VGG-like convolutional neural network as the shared encoder [30]. Auto-encoders consist of two primary components. We mitigate inaccurate feature detection by exploiting the inherent dimensionality reduction advantage of encoders. The network is designed to perform effectively across diverse image modalities.

Feature detection and description are initially applied to each modality, utilizing our previous work [29]. This approach enhances the detection of a maximal quantity of feature points.

#### 3.2.2. Enhancing Infrared and Visible Image Matching Using a Trade-Off between Key-Points Threshold and Non-Maximum Suppression

In this study, we present an augmented algorithm inspired by the Superglue framework [31], appropriate for matching common features in the IR and VIS images of the same scene. The proposed approach introduces a novel trade-off mechanism between the key-points threshold ($\tau_{\mathrm{key}}$) and non-maximum suppression (NMS) factor (α), providing a customizable balance between the quantity and quality of matched key points.

Effective image matching across modalities, such as IR and VIS, is critical for various applications, including image registration. The Superglue algorithm serves as a foundation for our work, with a focus on optimizing the matching process by introducing a parameterized trade-off.

The proposed method follows the Superglue pipeline with a notable modification in the matching stage. Let $K_{1}$ and $K_{2}$ be the key point sets extracted from IR and VIS images, respectively. The matching score ($S_{ij}$) between key point *i* in $K_{1}$ and key point *j* in $K_{2}$ is given by the following:

$$S_{ij}=\mathrm{cosine}\_\mathrm{similarity}\left(d_{i},d_{j}\right)\cdot \exp\left(-\frac{∥{\mathrm{pos}}_{i}-{\mathrm{pos}}_{j}{∥}^{2}}{2\sigma^{2}}\right)$$

where $d_{i}$ and $d_{j}$ are descriptors, ${\mathrm{pos}}_{i}$ and ${\mathrm{pos}}_{j}$ are positions, and σ is a smoothing parameter.

The key innovation lies in the adjustment of the key-points threshold ($\tau_{\mathrm{key}}$) and NMS factor (α). A lower $\tau_{\mathrm{key}}$ includes more key points, while α regulates NMS aggressiveness. Our trade-off is defined as follows:

$$\mathrm{Trade-off}=\tau_{\mathrm{key}}\times \alpha$$

This allows users to navigate a spectrum from highly selective (Trade-off ≈0) to more inclusive (Trade-off ≈1) matching strategies.

We have conducted experiments on a diverse dataset, varying $\tau_{\mathrm{key}}$ and α. The results demonstrate that our trade-off mechanism influences both the quantity and quality of matched key points, providing practitioners with a versatile tool for fine-tuning the matching process.

The proposed algorithm extends the capabilities of the Superglue framework by introducing a trade-off between the key-points threshold and NMS, offering a flexible approach for IR and VIS image matching.

### 3.3. Homography Estimation

Homography estimation is a critical component in aligning VIS and IR images for various applications. The proposed method, leveraging key points and a refined estimation process, provides an accurate and robust solution for achieving geometric correspondence between these modalities. This advancement contributes to enhancing the reliability of multi-modal image analysis in diverse scenarios. Given a set of corresponding key points $P_{\mathrm{VIS}}=\left\{\left(x_{{\mathrm{VIS}}_{1}},y_{{\mathrm{VIS}}_{1}}\right),\ldots ,\left(x_{{\mathrm{VIS}}_{n}},y_{{\mathrm{VIS}}_{n}}\right)\right\}$ in the VIS image and $P_{\mathrm{IR}}=\left\{\left(x_{{\mathrm{IR}}_{1}},y_{{\mathrm{IR}}_{1}}\right),\ldots ,\left(x_{{\mathrm{IR}}_{n}},y_{{\mathrm{IR}}_{n}}\right)\right\}$ in the IR image, the goal is to find the homography matrix *H* that transforms points from the VIS to the IR coordinate space.

The homography matrix *H* is computed using the Direct Linear Transform (DLT) algorithm [32]. Let $p_{{\mathrm{VIS}}_{i}}$ and $p_{{\mathrm{IR}}_{i}}$ represent the homogeneous coordinates of corresponding points in the VIS and IR images, respectively.

(1) $$p_{{\mathrm{IR}}_{i}}∼H\cdot p_{{\mathrm{VIS}}_{i}}$$

The estimation involves solving the linear system Ah=0, where *h* is the vectorized form of *H*. The matrix *A* is constructed from the coordinates of corresponding points.

(2) $$A=\left[\begin{array}{ccccccccc} -x_{{\mathrm{VIS}}_{1}} & -y_{{\mathrm{VIS}}_{1}} & -1 & 0 & 0 & 0 & x_{{\mathrm{VIS}}_{1}}x_{{\mathrm{IR}}_{1}} & y_{{\mathrm{VIS}}_{1}}x_{{\mathrm{IR}}_{1}} & x_{{\mathrm{IR}}_{1}}\\ 0 & 0 & 0 & -x_{{\mathrm{VIS}}_{1}} & -y_{{\mathrm{VIS}}_{1}} & -1 & x_{{\mathrm{VIS}}_{1}}y_{{\mathrm{IR}}_{1}} & y_{{\mathrm{VIS}}_{1}}y_{{\mathrm{IR}}_{1}} & y_{{\mathrm{IR}}_{1}}\\ \vdots & \vdots & \vdots & \vdots & \vdots & \vdots & \vdots & \vdots & \vdots \\ -x_{{\mathrm{VIS}}_{n}} & -y_{{\mathrm{VIS}}_{n}} & -1 & 0 & 0 & 0 & x_{{\mathrm{VIS}}_{n}}x_{{\mathrm{IR}}_{n}} & y_{{\mathrm{VIS}}_{n}}x_{{\mathrm{IR}}_{n}} & x_{{\mathrm{IR}}_{n}}\\ 0 & 0 & 0 & -x_{{\mathrm{VIS}}_{n}} & -y_{{\mathrm{VIS}}_{n}} & -1 & x_{{\mathrm{VIS}}_{n}}y_{{\mathrm{IR}}_{n}} & y_{{\mathrm{VIS}}_{n}}y_{{\mathrm{IR}}_{n}} & y_{{\mathrm{IR}}_{n}} \end{array}\right]$$

The homography matrix *H* is obtained by finding the null space of *A*. Subsequently, *H* is refined using a non-linear optimization method, such as Levenberg–Marquardt, to improve accuracy.

### 3.4. Infrared and Visible Image Fusion Block

Employing the advancements of deep learning as a powerful tool in various image-processing applications, we applied a deep-learning-based image fusion method [33] in this study to seamlessly integrate features from IR and visible imagery. As Figure 2 indicates, this method encompasses a series of steps, starting with the decomposition of the source images into base components and detailed content [34]. Subsequently, the fusion process begins with the integration of base parts utilizing a weighted-averaging strategy. Simultaneously, the detailed content undergoes fusion through a multi-layer approach facilitated by a deep learning network [30]. The culmination of these steps results in the reconstruction of a fused image that provides a comprehensive representation, amalgamating information from both modalities.

## 4. Results and Discussion

This section thoroughly examines the results derived from this study, investigating key findings and their consequential implications. This research focused on enhancing image registration and fusion for non-destructive evaluation of infrastructure, utilizing a dual-sensor approach with infrared thermography and visible images. Throughout the experiments, various factors and variables were investigated. This section presents an overview of these results, beginning with a multi-scale template matching approach. Subsequently, an in-depth discussion of feature point detection and matching for both infrared and visible images is embarked upon, focusing on a trade-off between non-maximum suppression and feature-point threshold and concluding with insights into image registration and fusion. Finally, conclusions are drawn, and insights into the broader implications of our research are provided through the experiments and findings. A more profound understanding of image registration and fusion using multi-sensors for advanced infrastructure visualization is provided.

### 4.1. Dataset and Implementation Details

**Figure 3:**
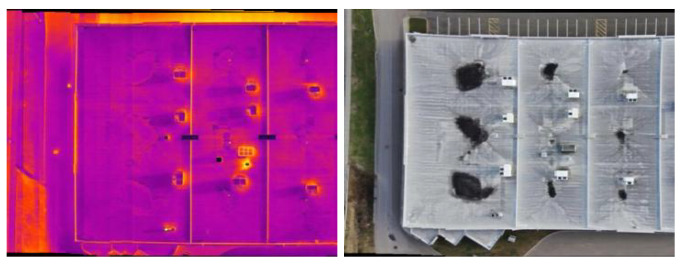
The **right** image is the visible image, and the **left** one is its infrared counterpart of the same scene.

This study used a dataset containing coupled thermal and visible images of industrial assets to evaluate the proposed approach. The dataset is related to the roof of a building. This dataset includes 73 couple thermal and visible images. A DJI M300 drone equipped with a Zenmuse H20T camera (Da-Jiang Innovations Science and Technology Co., Ltd., Shenzhen, China) was employed for acquiring thermal and visible images at an altitude of 15 m. Figure 3 shows some samples of this study, and Table 1 outlines the specifications of both the infrared and visible cameras. All the experiments were implemented in the Python3.9 programming language on a computer with a 2080Ti GPU.

### 4.2. Results on Multi-Scale Template Matching

This section presents the detailed results of the application of the multi-scale template matching approach aimed at exploring the common regions within infrared and visible images. The images showcased in Figure 3 serve as an example, which result from the image stitching process.

**Figure 4:**
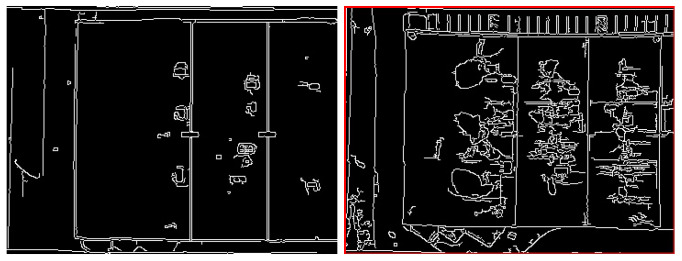
Canny edge representations of thermal and visible images: The **left** image is the canny edge representation of thermal (template) image. The **right** image is the canny edge representation of the visible image.

As shown in Figure 3, the infrared image has a smaller field of view. Therefore, it is considered the template. Notably, in Figure 4, the edges in both the infrared and visible images share similarities. It leads to extracting Canny edges from the template and the counterpart visible image. Figure 4 indicates the Canny edge map representation of the infrared image, which is used as a template, and the visible image sample.

The algorithm is multi-scale since it runs iteratively. At each iteration, the image resizes, and the Canny edge map is computed. Subsequently, template matching, facilitated by the cv2.matchTemplate module in Python, is employed to identify the (x, y)-coordinates of the image with the most significant correlation coefficient. To maintain a record of these values, a variable is employed. After the algorithm, a meticulous analysis is conducted to determine the (x, y)-coordinates corresponding to the region with the most substantial correlation coefficient response across all scales.

**Figure 5:**
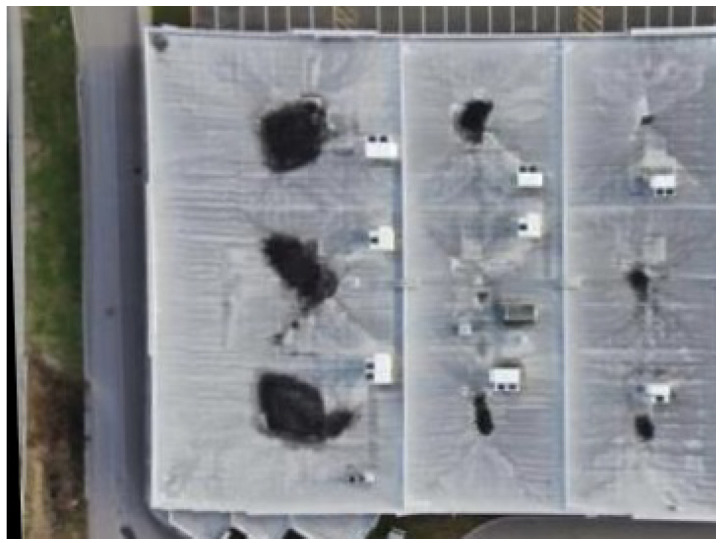
The result of the multi-scale template matching algorithm. The shared region between IR and VIS images is found, and the related area in the VIS image is cropped.

In the final step, through the previous detailed information, the shared region of the images is cropped precisely. This process ensures a thorough exploration of the common regions between the infrared and visible images, offering valuable insights into the nuances of multi-scale template matching for image fusion in infrastructure visualization. Figure 5 shows the shared region of the infrared and visible images.

### 4.3. Infrared and Visible Feature Detection and Matching

This section presents the feature detection and matching results conducted on a visible image and its infrared counterpart. Initially, feature detection was performed utilizing the self-supervised learning approach for each infrared and visible image using the method introduced in [26]. Consequently, many high-quality and high-quantity features were detected across both image modalities depicting the same scene.

Despite the abundance of high-quality and high-quantity features detected in each modality, the significant disparities between each imaging modality necessitated exploring alternative approaches to enhance the feature-matching phase. To address this challenge, experiments were conducted to trade off between non-maximum suppression (NMS) and feature threshold. Subsequently, the ensuing results are comprehensively delineated and analyzed in the subsequent sections.

In the ensuing subsections, the effect of feature-point threshold and non-maximum suppression on infrared and visible feature matching is investigated, individually. Also, a detailed analysis of the results is provided. Subsequently, the challenges encountered are introduced, and the hypothesis behind employing a trade-off between feature-point threshold and NMS for feature matching is explored.

Careful optimization of feature detection parameters is important for achieving high-quality and high-quantity features for image registration. According to the existing literature [31], default values for feature-point threshold and non-maximum suppression (NMS) are commonly set to 0.05 and 5, respectively. This paper provides a deeper investigation conducted into the effect of these parameters on feature detection and subsequent matching in the context of infrared (IR) and visible (VIS) image registration.

**Figure 6:**
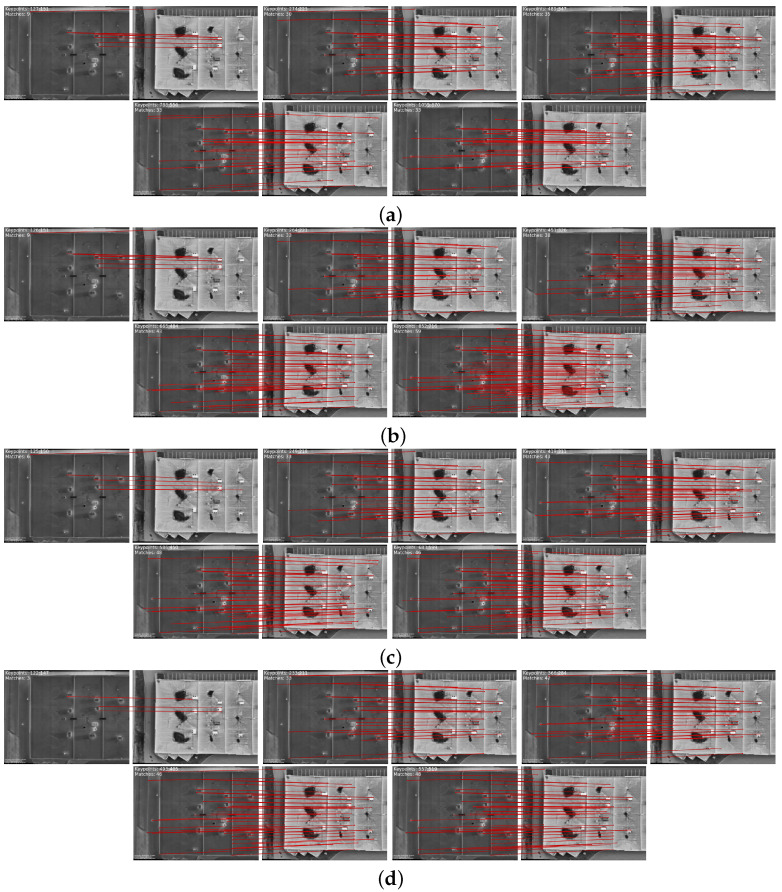
Feature matching results using proposed method with the trade-off between NMS and feature-points threshold parameters: (**a**) NMS:5, key-points threshold of each image set from left to right: 0.05, 0.005, 0.0005, 0.00005, 0.00000. (**b**) NMS:6, key-points threshold of each image set from left to right: 0.05, 0.005, 0.0005, 0.00005, 0.00000. (**c**) NMS:7, key-points threshold of each image set from left to right: 0.05, 0.005, 0.0005, 0.00005, 0.00000. (**d**) NMS:8, key-points threshold of each image set from left to right: 0.05, 0.005, 0.0005, 0.00005, 0.00000.

Figure 6a illustrates the effect of decreasing the feature-point threshold while keeping NMS constant at 5. As the threshold approaches zero, a notable increase in the quantity of detected feature points is observed. As depicted in Figure 6 and Table 1, while a higher quantity of features may seem beneficial at first glance, it becomes evident that this increase introduces false matches during feature matching. These false matches can significantly impair the accuracy and reliability of image registration, as they lead to erroneous correspondences between points in the IR and VIS images. The adjustment of the NMS value is explored to address this challenge and enhance the quality of feature points, thereby fostering less faulty feature matching. By investigating NMS from 5 to 8, redundant feature points are effectively eliminated, as depicted in Figure 6. This adjustment aims to mitigate the adverse effects of redundancy in feature points on the overall registration process.

The investigation into the elimination of redundant feature points through NMS adjustment is not just a theoretical exploration but a significant step forward in the field of image registration. It is motivated by the hypothesis that reducing redundancy will lead to more promising feature matching and subsequently improve the accuracy of image registration. By refining the selection of salient feature points and eliminating redundant features, a reduction in redundant or false matches and an enhancement in feature-matching quality are anticipated in the experiments, thereby contributing to the advancement of image registration techniques.

As depicted in Figure 6 and detailed in Table 2, in each step of the key-point threshold increasing NMS, a gradual decrease is observed in the number of detected key points. However, despite this reduction, Table 3 indicates it is noteworthy that the number of matching points either remains constant or exhibits an increase. This trend suggests the successful optimization of the feature-point detection process alongside the strategic management of non-maximum suppression (NMS) parameters. The balance achieved between feature-point optimization and NMS is underscored by these findings, indicating a highly effective trade-off. By selectively retaining only the most relevant key-points, the precision and discriminative power of feature matching are enhanced while maintaining spatial correspondence and minimizing computational overhead.

### 4.4. Infrared and Visible Image Registration

This section indicates the visible images aligned based on the template thermal image using the approach discussed in the previous section. In IR and VIS image registration, we undertook a comprehensive investigation with a precise optimization of the feature-matching process. Non-maximum suppression and key-point thresholding were methodically varied to evaluate their effects on the quantity and quality of detected feature points.

**Figure 7:**
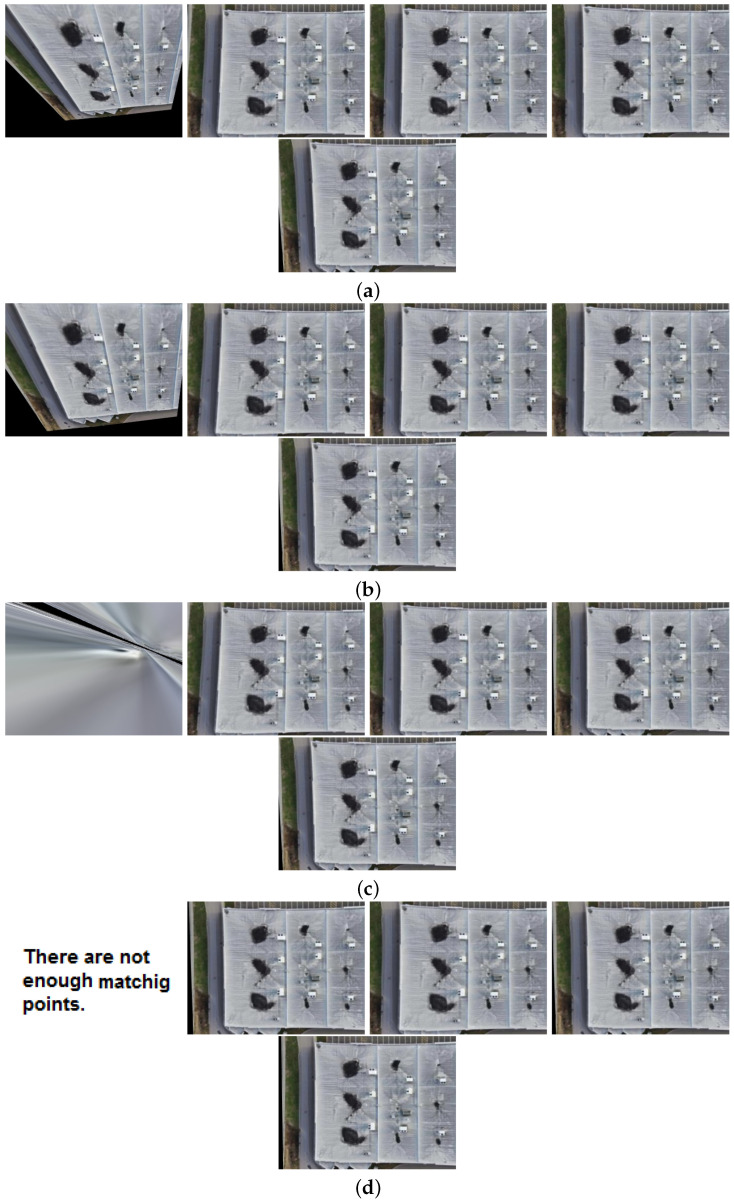
Image registration results using proposed method with the trade-off between NMS and feature-points threshold parameters: (**a**) NMS:5, key-points threshold of each image set from left to right: 0.05, 0.005, 0.0005, 0.00005, 0.00000. (**b**) NMS:6, key-points threshold of each image set from left to right: 0.05, 0.005, 0.0005, 0.00005, 0.00000. (**c**) NMS:7, key-points threshold of each image set from left to right: 0.05, 0.005, 0.0005, 0.00005, 0.00000. (**d**) NMS:8, key-points threshold of each image set from left to right: 0.05, 0.005, 0.0005, 0.00005, 0.00000.

Figure 7 illustrates the experimentation included a spectrum of NMS values ranging from 5 to 8, coupled with corresponding key-point threshold values ranging from 0.05 to 0.00000. As depicted in Figure 7, the results clarify the effect of varying NMS and key-point threshold parameters on image registration. Increasing NMS values correlated with a rise in detected feature points. Similarly, reducing the key-point threshold yielded a higher quantity of detected feature points. This observation underscores the sensitivity of feature detection to parameter adjustments, with modifications in NMS and key-point threshold directly influencing the density of identified key-points.

Furthermore, the analysis unveils a discernible trend concerning the interplay between NMS, key-point threshold, and the accuracy and confidence of detected feature points. As NMS values escalate, there is a demonstrable enhancement in the accuracy and confidence of feature points. This phenomenon suggests that heightened non-maximum suppression leads to selecting more robust and prominent key points, thereby bolstering the reliability of feature matching.

Summarizing the methodical exploration of NMS and key-point threshold parameters, our findings offer invaluable insights into their impact on feature matching performance in IR and VIS image registration. These observed trends underscore the criticality of parameter optimization in attaining optimal results. Notably, elevated NMS values contribute to amplified accuracy and confidence in detected feature points. These significant findings represent a noteworthy contribution to the domain of image registration and lay the groundwork for further advancements in feature matching techniques across diverse application scenarios.

In image registration, the trade-off of the key-point threshold and NMS presents a robust strategy for achieving precise alignment between infrared and visible images.

Figure 7 indicates the sensitivity facilitated by a near-zero key-point threshold enables the detection of fine details and subtle features, contributing to the accuracy of correspondences identified during the registration process. Simultaneously, the increased selectivity afforded by intensified NMS ensures that only the most salient key points are retained, minimizing the influence of noise and irrelevant information. Therefore, in Figure 7, in every step (a) to (d) with decreasing key-points threshold and increasing NMS, the aligned image is more and more formable.

Therefore, the registration algorithm can effectively align the images, accounting for geometric and radiometric disparities while preserving essential structural information. The seamless fusion of infrared and visible images is facilitated by the precise alignment achieved through parameter optimization.

### 4.5. Results on Infrared and Visible Image Fusion

Finally, this subsection presents the fusion of infrared and visible images to produce images with enhanced information content. Through the fusion algorithm, we aim to use the complementary strengths of both modalities to provide comprehensive insights into the target scene.

The successful fusion of visible and infrared images is a theoretical concept and a crucial tool for various real-world applications, including infrastructure inspection. This section delves into the practical results of the fusion of these two modalities and examines how the aligning process enhances data visualization, making the research directly applicable to the audience’s work.

**Figure 8:**
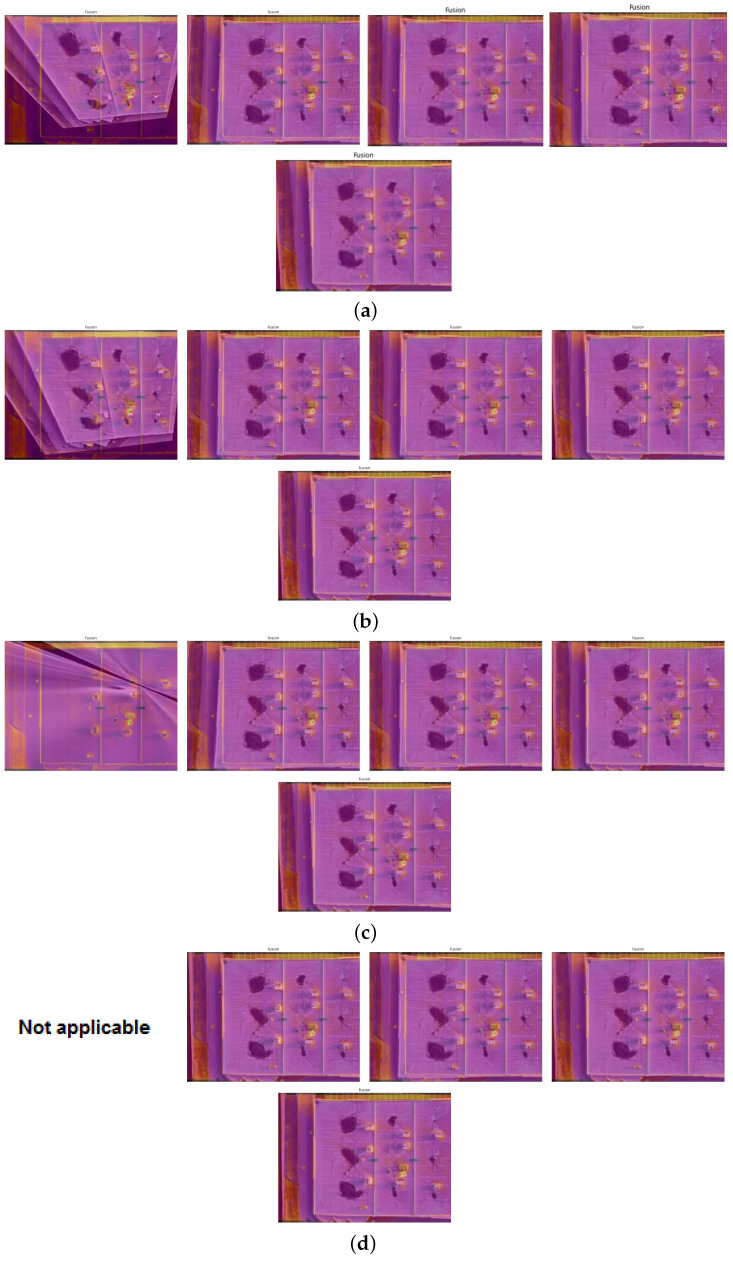
IR and VIS image fusion results using the proposed method: (**a**) NMS:5, key-points threshold of each image set from left to right: 0.05, 0.005, 0.0005, 0.00005, 0.00000. (**b**) NMS:6, key-points threshold of each image set from left to right: 0.05, 0.005, 0.0005, 0.00005, 0.00000. (**c**) NMS:7, key-points threshold of each image set from left to right: 0.05, 0.005, 0.0005, 0.00005, 0.00000. (**d**) NMS:8, key-points threshold of each image set from left to right: 0.05, 0.005, 0.0005, 0.00005, 0.00000.

The fusion samples depicted in Figure 8 visually confirm the alignment’s effectiveness. The fusion of visible and infrared data in these samples demonstrates the potential of the fusion process to enhance data visualization and facilitate comprehensive analysis. The infrared template image is fused using each aligned image provided by the previous subsection. Figure 8 illustrates the effectiveness of the key-point threshold and non-maximum suppression trade-off optimization. The most accurate fusion is related to the image with nms = 8 and feature-point threshold = 0.00000.

An evaluation metric is employed to assess the quality of alignment. This study chooses the Euclidean distance metric due to its simplicity and effectiveness in measuring the spatial discrepancy between corresponding points. The alignment accuracy can be determined by calculating the Euclidean distance between the points identified in the reference and registered images. Table 3 shows the Euclidean distance for the best alignment is equal to 0.37, which is related to the image with nms = 8 and feature-point threshold = 0.00000.

The results of the alignment evaluation reveal the efficacy of the proposed method. Across the sample images, the Euclidean distance values consistently indicate precise alignment, even under challenging conditions. This validation underscores the robustness of the alignment algorithm and its suitability for other real-world applications.

In conclusion, aligning visible and infrared images is critical in various fields, including remote sensing and infrastructure inspection. The proposed method achieves precise alignment through meticulous computational techniques and rigorous evaluation, laying the groundwork for advanced data fusion and analysis.

### 4.6. Evaluation Metrics

The alignment accuracy was evaluated using the Euclidean distance metric, which involves calculating the distance between two feature points in the image by taking the square root of the sum of the squared differences between their coordinates. The results for our case studies are presented in Table 4.

The process involved determining the corresponding point in the registered images for each point of interest identified in the reference image, followed by calculating the Euclidean distance between these paired points.

For the assessment detailed in Table 3, the process involved (1) selecting the most challenging point from the reference image, (2) locating its counterpart in the registered images, and (3) computing the Euclidean distance between these points across all images sequentially. The findings indicate precise alignment, with Euclidean distance values reflecting accuracy for the given parameters of NMS = 8 and key-point threshold = 0.00000.

## 5. Conclusions

In this study, we encountered initial challenges related to the differences in field of view between infrared and visible sensors. To address this, a multi-scale template matching approach was implemented, facilitating the alignment and correlation of data captured by sensors with varying fields of view. This technique ensures accurate information despite the disparities in sensor perspectives.

Another critical challenge we faced was aligning the images from both modalities to optimize the fusion of infrared and visible images. We introduced a novel and robust image registration approach to overcome this obstacle. This method enhances the feature detection phase and feature matching phase through a trade-off between non-maximum suppression and key-points threshold using a self-supervised auto-encoder for the feature detection and description phase and a graph neural network for feature matching phase for infrared and visible image registration. By employing this innovative strategy, we aim to achieve precise alignment, facilitating seamless integration of infrared and visible data and mitigating challenges associated with sensor misalignment. It contributes significantly to the success of the image fusion process.

Lastly, we employed a robust multi-modal image fusion technique to ensure a comprehensive fusion of image modalities. Through these concerted efforts, we have addressed key challenges in sensor integration and alignment, paving the way for more accurate and reliable image fusion techniques in advanced applications.

In future work, addressing the limitations related to sensor types would be a valuable extension of the proposed IR and VIS image registration technique. Different sensors have varying resolutions, sensitivities, and spectral ranges, which can affect the accuracy and reliability of image registration. By developing methods to effectively handle data from a wider range of sensor types, including those with different specifications and performance characteristics, the technique could become more robust and versatile. This improvement would enhance its applicability across diverse fields such as remote sensing and medical imaging, where different sensor technologies are commonly used. In remote sensing, the technique could enhance disaster monitoring and precision farming by integrating various spectral images. In medical imaging, it could improve diagnostic accuracy by fusing different modalities like MRI, CT, and PET scans. These extensions underscore the technique’s versatility and broad applicability beyond infrastructure inspection.

## Figures and Tables

**Table 1:** Specifications of visible and infrared cameras mounted on the drone.

Specification	Visible Camera (Zenmuse H20)	IR Camera (Zenmuse H20T)
**Dimensions**	150 × 114 × 151 mm	167 × 135 × 161 mm
**Storage Temperature**	−20° to 60 °C	
**Resolution**	5184 × 3888	640 × 512
**Lens**	DFOV: 66.6°	DFOV: 40.6°

**Table 2:** Feature-points quantity comparison for NMS and feature-point threshold trade-off. Infrared image feature quantity: Visible image feature quantity.

NMS	a = 5	b = 6	c = 7	d = 8
0.05	127:151	126:151	125:150	122:146
0.005	274:223	246:221	249:218	233:211
0.0005	489:347	451:326	419:311	366:284
0.00005	388:566	665:484	586:450	493:405
0.00000	1055:810	825:716	683:599	537:519

**Table 3:** Feature-matching comparison for NMS and feature-point threshold trade-off.

NMS	a = 5	b = 6	c = 7	d = 8
0.05	9	9	6	3
0.005	30	33	33	33
0.0005	35	38	43	42
0.00005	33	43	48	46
0.00000	33	59	46	48

**Table 4:** The alignment accuracy of the proposed method using the Euclidean distance metric.

	NMS = 5	NMS = 6	NMS = 7	NMS = 8
0.05	-	-	-	-
0.005	2.64	2.64	37	2.64
0.0005	2.64	2.64	37	2.64
0.00005	2.64	1.57	3.4	1.13
0.00000	3.40	1.13	1.13	**0.37**

## Data Availability

Data is contained within the article.

## Abbreviations

The following abbreviations are used in this manuscript:

NMS	Non-Maximum Suppression
IRT	Infrared Thermography
NDE	Non-Destructive Evaluation
NDT	Non-Destructive Testing
AKAZE	Accelerated Keypoint and Zernike Moment Descriptor
ORB	Oriented FAST and Rotated BRIEF
SIFT	Scale-Invariant Feature Transform
DLT	Direct Linear Transfer
